# Proceedings of the Twenty-third Annual Meeting of the Homœopathic Medical Society of Ohio

**Published:** 1888-04

**Authors:** 


### BOOK NOTICES AND REVIEWS.

PROCEEDINGS OF THE TWENTY-THIRD ANNUAL MEETING OF THE HOMŒOPATHIC MEDICAL SOCIETY OF OHIO. Drs. C. E. Walton, H. Pomeroy, and N. Schneider, Publication Committee. 1887.

The twenty-third annual meeting of the Homœopathic Medical Society of Ohio was begun May 10th, 1887, at Cleveland. The Publication Committee send out this neat volume of three hundred and eight pages to show what the homœopaths of Ohio are doing. The papers and their discussion cover a great variety of subjects and are interesting, but, as usual, the most important branch of medical knowledge is slighted. We refer to the Materia Medica.

